# Severe ampullary angiodysplasia bleeding: precautionary biliopancreatic stenting enables safe and effective argon plasma coagulation hemostasis

**DOI:** 10.1055/a-2988-8301

**Published:** 2026-11-03

**Authors:** Maria Terrin, Marco Spadaccini, Mario Ricchiuti, Lorenzo Meli, Alessandro Fugazza, Cesare Hassan, Alessandro Repici

**Affiliations:** 1 Division of Gastroenterology and Digestive Endoscopy 551905 IRCCS Humanitas Research Hospital Department of GastroenterologyRozzano, MilanoLombardiaItaly; 2 Department of Biomedical Sciences 551905 Humanitas UniversityPieve EmanueleLombardiaItaly; 3 Department of Biomedical Sciences 437807 Humanitas UniversityPieve EmanueleLombardiaItaly; 4 Department of Clinical Medicine and Surgery 478484 Università degli Studi di Napoli Federico II Scuola di Medicina e ChirurgiaNapoliCampaniaItaly; 5 Department of Medical Science and Public Health 3111 University of CagliariMonserratoCagliariItaly

Angiodysplasia is the most common vascular abnormality of the gastrointestinal (GI) tract, accounting for ~5% of non-variceal upper GI bleeding (UGIB) 1​ ​2​ . However, involvement of the major duodenal papilla as the bleeding source is exceedingly rare, with only a few reports. 2​ ​3​ Owing to the proximity of the papillary orifices, the optimal endoscopic treatment remains undefined, as any intervention carries a significant risk of adverse events (AEs), such as pancreatitis or ductal injury/stricture 4​ ​5​ .

A 71-year-old man without a previous history of GI endoscopy presented with melena and severe anemia (Hb 5.4 g/dL), complicated by myocardial injury. After resuscitation, urgent esophagogastroduodenoscopy revealed active bleeding from an angiodysplastic lesion of the major duodenal papilla ( Fig. 1 ).

**Fig. 1:**
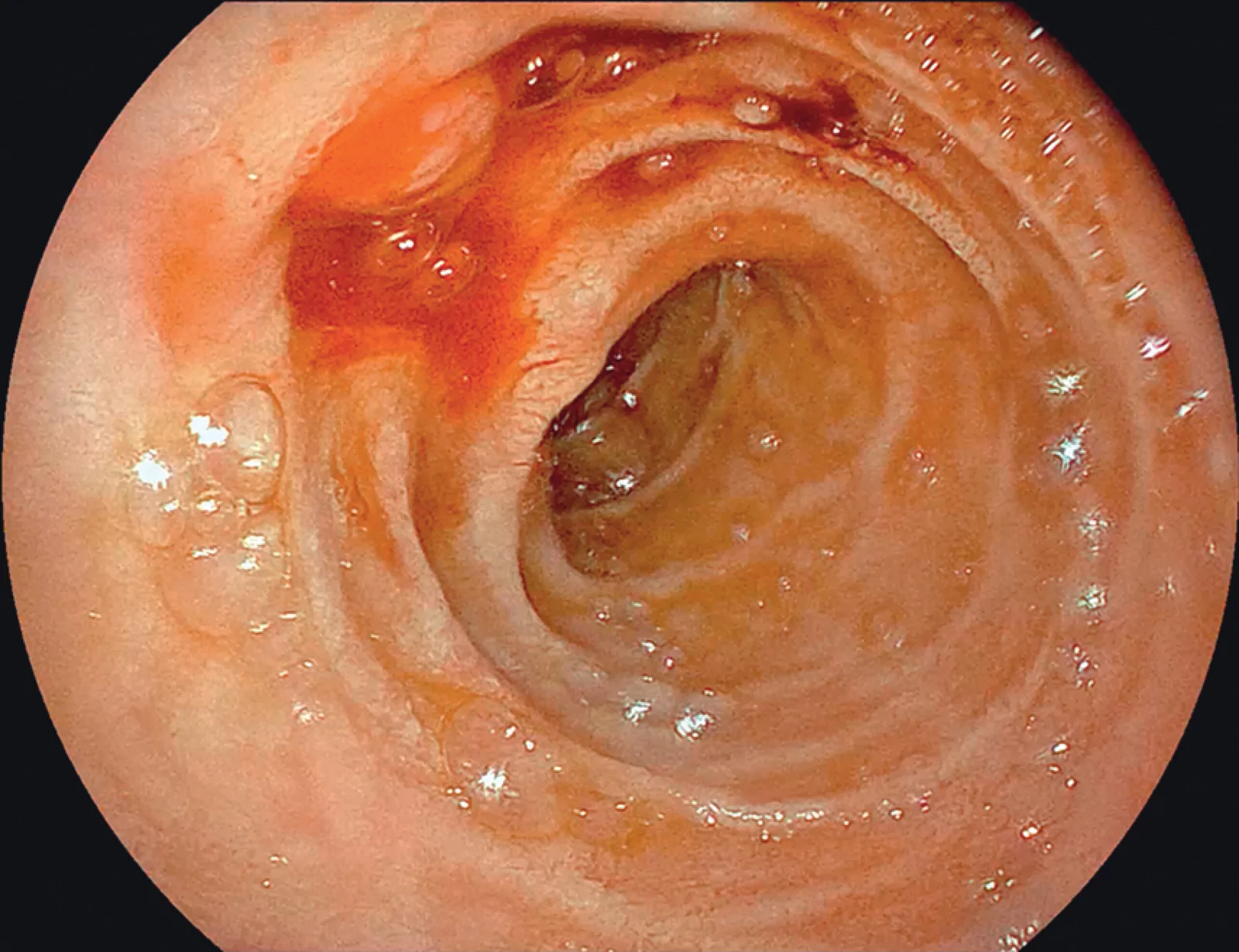
An endoscopic view of the major duodenal papilla showing active bleeding from an angiodysplastic lesion, obtained with a forward-viewing standard gastroscope (Fujifilm ELUXEO 700 Series, model EG-760R, with a distal end diameter of 9.3 mm and a working channel of 2.8 mm).

Periampullary epinephrine injection failed to achieve hemostasis. Thermal treatment was then planned; however, to avoid thermal injury to the pancreatobiliary ducts, endoscopic retrograde cholangiopancreatography (ERCP) with prophylactic biliary and pancreatic stent placement was performed ( Figs. 2 3 ).

**Fig. 2:**
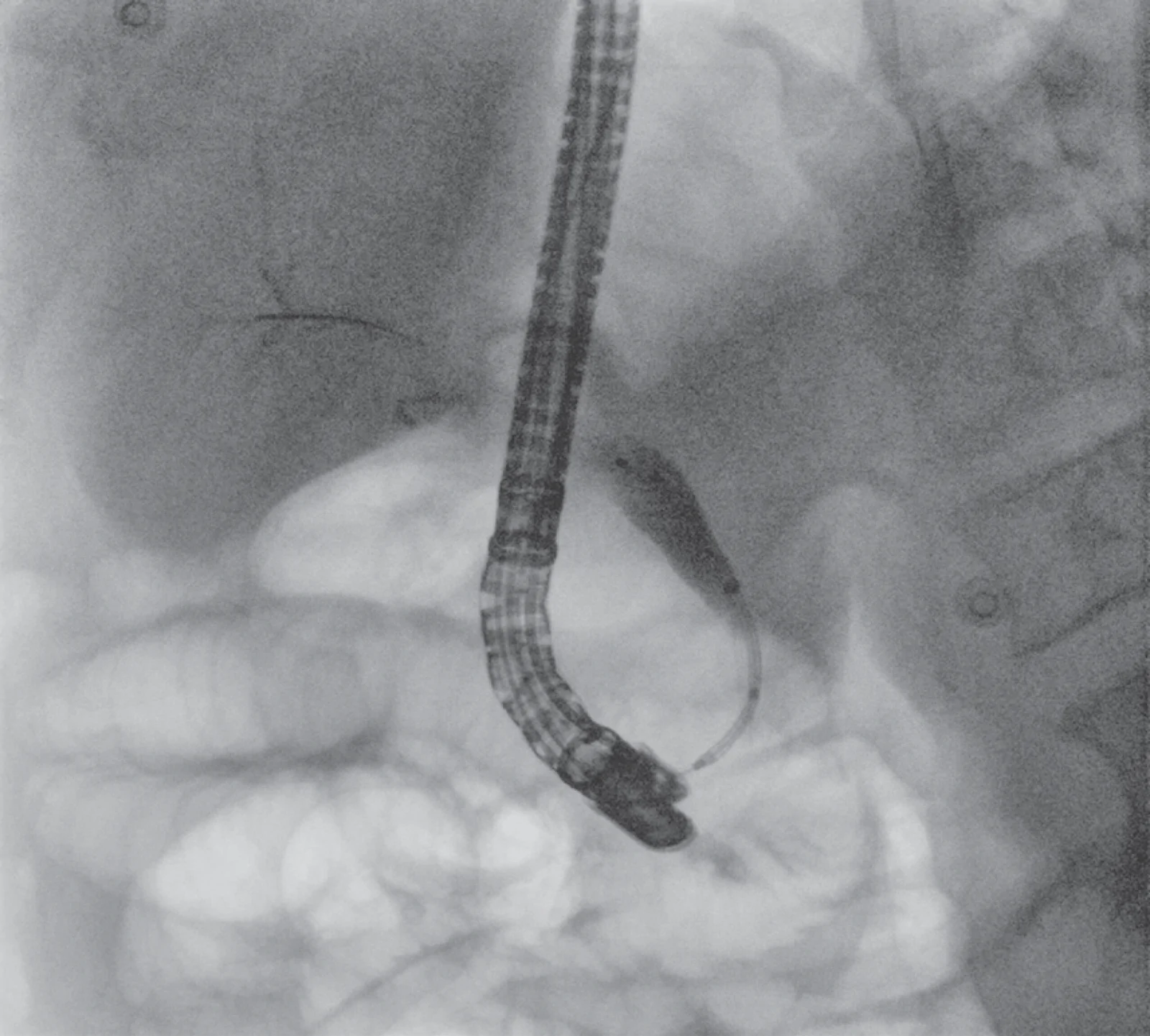
A fluoroscopic view of the biliary tree showing the pancreatic plastic stent and the fully covered biliary self-expandable metal stent (FC-SEMS) in place *.*

**Fig. 3:**
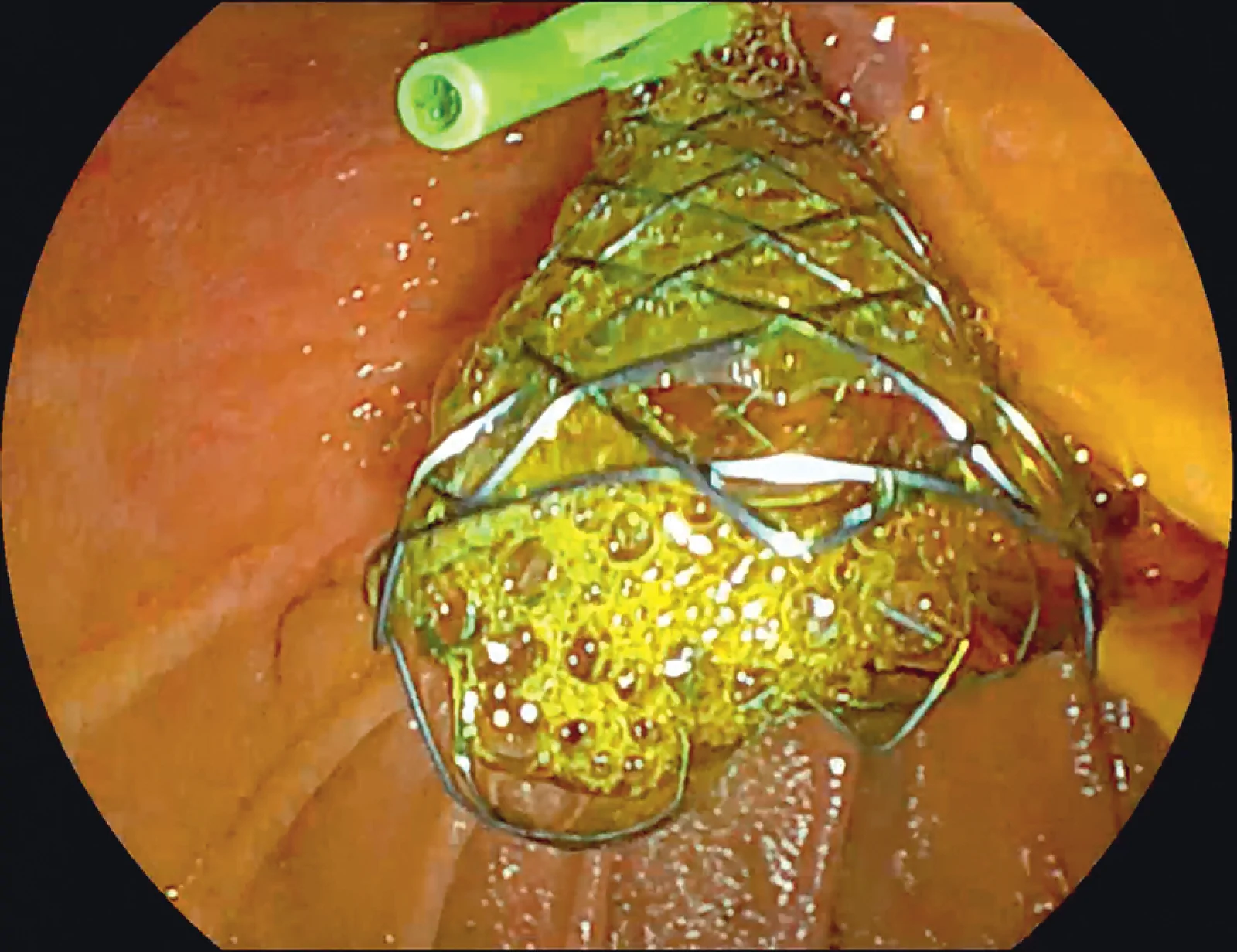
An endoscopic view of the major duodenal papilla with the pancreatic plastic stent and the biliary FC-SEMS in situ (Fujifilm, model ED-580XT, with a distal end diameter of 13.1 mm and a working channel of 4.2 mm). All procedures were performed with the patient in the left lateral decubitus position under anesthesiologist-assisted sedation with spontaneous ventilation. A 5 Fr×5 cm plastic pancreatic stent and a 10 mm×4 cm FC-SEMS were deployed under fluoroscopic guidance *.*

Complete hemostasis was then successfully achieved with argon plasma coagulation (APC; **Video 1** , Fig. 4 ).

**Video 1** Hemostatic treatment using a single-use FiAPC probe (2200 A, 2.3 mm, straight fire; ERBE). Unlike conventional hemostatic devices, the frontal APC probe allows optimal adaptation to the duodenoscope.


**Fig. 4:**
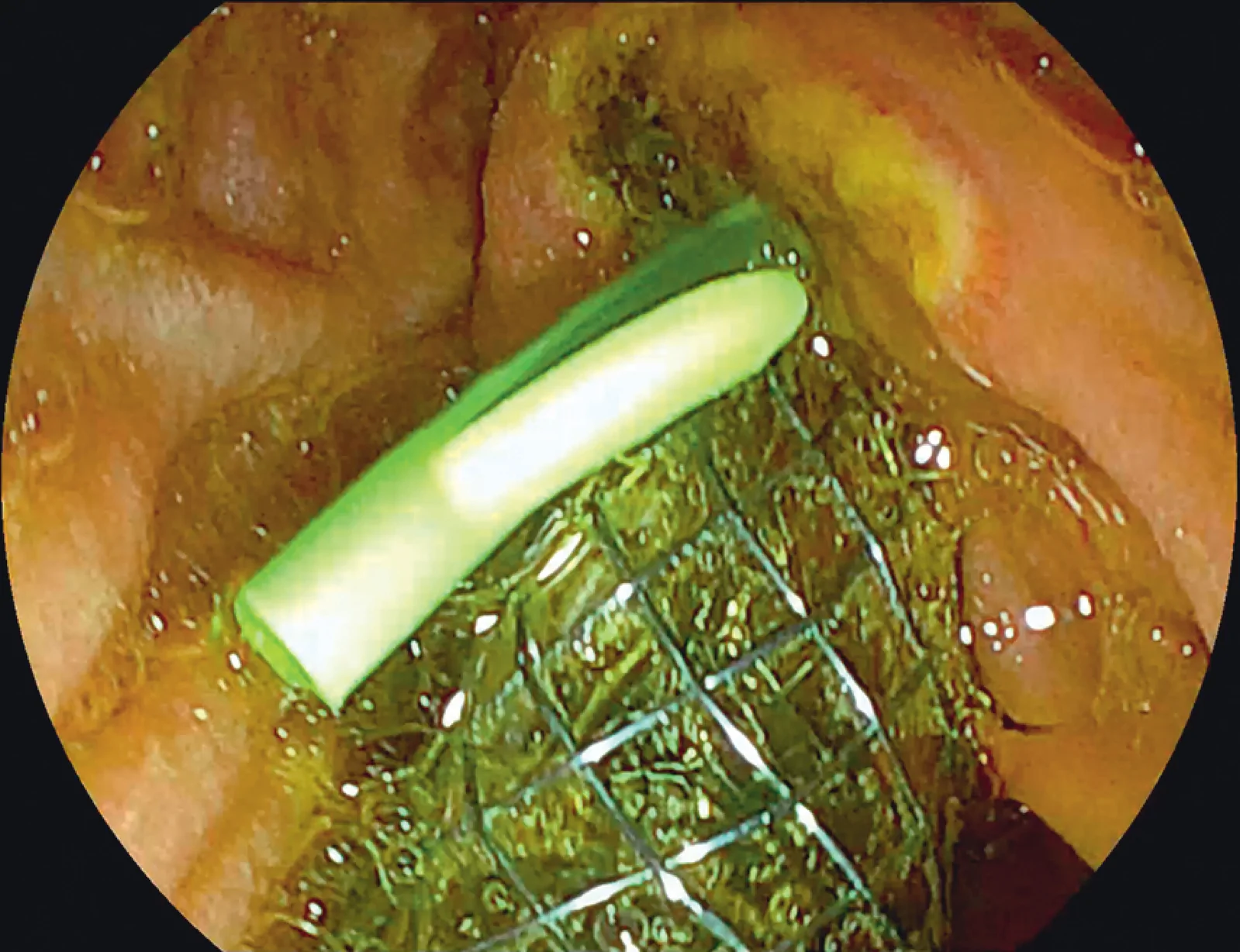
An endoscopic view after successful APC hemostasis, with pancreatic stent and biliary FC-SEMS in situ.

No ERCP-related AEs occurred. At 2-month follow-up, the patient remained asymptomatic. Repeat endoscopy showed healing of the treated area ( Fig. 5 ), and both stents were removed.

**Fig. 5:**
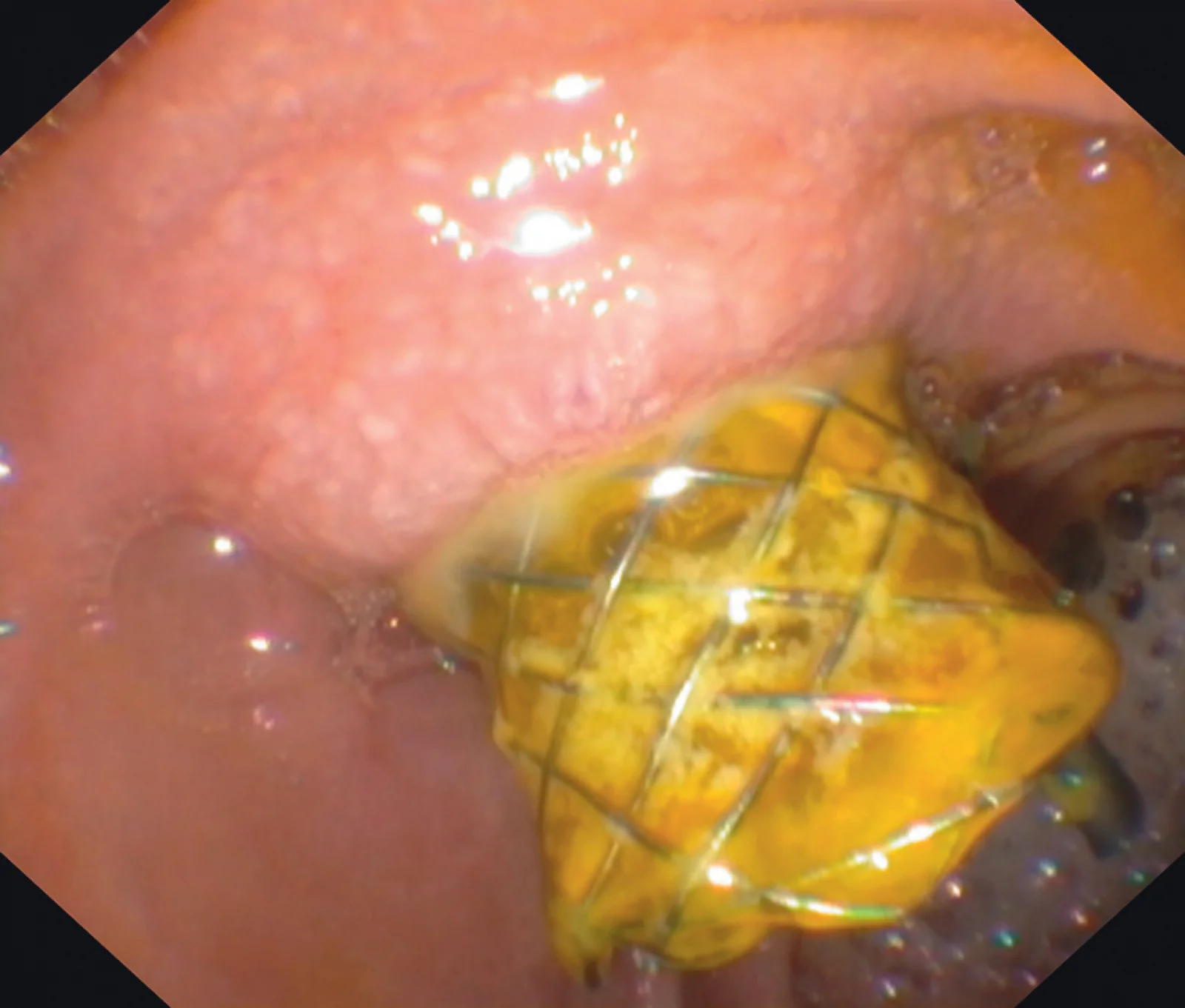
An 8-week follow-up endoscopic view showing pancreatic stent and biliary FC-SEMS in situ, with no evidence of rebleeding *.*

Ampullary angiodysplasia is a rare but potentially life-threatening cause of UGIB. Its location makes standard hemostatic approaches challenging and potentially hazardous. 3​ ​4​ Thermal hemostatic treatment may represent a viable option, particularly when coupled with prophylactic stenting, balancing the need for prompt intervention against the risk of severe AEs. 1​ ​2​ 4​​ ​5​ A key lesson illustrated by this case is the importance of cross-disciplinary expertise for endoscopists performing emergencies, as successful management was made possible by the awareness of the specific challenges posed by the anatomical site of bleeding and the availability of on-call pancreatobiliary endoscopists. This case contributes to the limited body of literature on this field, suggesting a prophylactic ERCP-based approach.

Endoscopy_UCTN_Code_TTT_1AO_2AD

## Declaration of GenAI Use

The authors confirm that no generative artificial Intelligence was used in the preparation of this manuscript
